# The global surgical triple goal: Surgery must improve health, be equitable, and environmentally sustainable

**DOI:** 10.1016/j.joclim.2025.100458

**Published:** 2025-06-07

**Authors:** Rhea Liang, John W. Orchard, Stephen J. Robson

**Affiliations:** a Department of Surgery, Gold Coast Health, 1 Hospital Boulevard, Southport QLD 4215, Australia; b Faculty of Health Sciences, Bond University, 14 University Dr, Robina QLD 4226, Australia; c School of Public Health, University of Sydney, A27 Fisher Rd, University of Sydney NSW 2050, Australia; d ANU College of Health and Medicine, 54 Mills Road, Acton ACT 2601, Australia

**Keywords:** Surgical procedures, Health equity, Sustainability, Climate change, Healthcare disparities, Resource-limited settings

## Abstract

If carbon emissions are not substantially reduced, human health will suffer irreparably. At the same time, global healthcare suffers from inequitable distribution which also leads to avoidable suffering. Health practitioners have always assessed treatment through the lens of potential benefits versus potential risks and harms. This lens must be widened to include global equity and carbon emissions if the triple goal of improving health, equitable delivery and environmental sustainability is to be achieved. This perspective looks at the surgery-specific challenges to sustainability through an equity lens, especially the tension between surgery being one of the most carbon-intensive forms of healthcare, and in many cases also being lifesaving or life-altering and hence essential. Carbon footprints should be made more visible, and sustainability should drive surgical innovation and be part of accreditation processes. Environmental impact should be built into research proposals, and the evidence arising from research used to make hard decisions about low value care. Carbon offsets may be a temporary measure. The surgery of the future must have a lower carbon footprint and consist of procedures that have been proven to be essential and/or of great benefit. Additionally, the burdens of achieving sustainable surgery must not exacerbate existing current or future healthcare inequity globally.

## Graphical abstract

**Figure:**
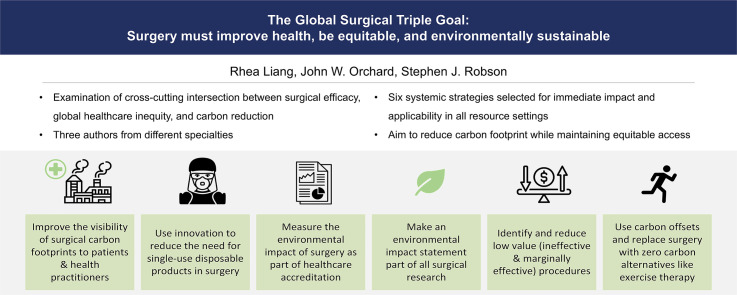

## 1 Introduction

Despite extraordinary technical advances in surgery, the goal of surgical care has remained unchanged throughout history: to alleviate human suffering and make people’s lives better [1]. Surgery is fundamental to health care and there is no prospect of this changing. Globally, almost 250 million operations are performed every year [2]; in the United Kingdom alone almost 8 million surgical procedures are performed yearly accounting for one-third of all hospital admissions [3]. Despite this, billions of people in low- and middle-income countries lack access to essential surgery [[4], [5], [6]] with estimates of unmet need as high as 143 million surgical procedures [7,8].

### 1.1 Climate change and global human health

While climate change remains the subject of political debate, the available data reveal that global warming is established, and that anthropogenic emissions of CO_2_ and other greenhouse gases are largely responsible for this warming [9,10]. Emissions of greenhouse gases resulting from human activity continue to increase, and gains in energy efficiency have been outpaced by increases in population and economic activity such as industrial processes [9]. These changes have a major effect on human health at a global level due to heat waves, droughts, and increased frequency and violence of major weather events [9,11,12]. As well, climate change is already accelerating monumental losses of biodiversity, and promoting the spread of infectious diseases such as malaria and gastrointestinal infections [13].

If surgeons are to fulfill their ethical obligation to safeguard health, then the consequences of providing health care must be important to us. As Lenzen and colleagues [14] point out:

“As investment in health care increases around the world, there is considerable potential for increasing harm to health from pollution and environmental change. People who are harmed by the environmental footprint of health care often live far away from those who benefit from the health care provided. Hence, doctors and other health sector leaders have a practical and ethical responsibility to measure, monitor, and address the environmental footprint of health care.”

It is estimated that health care is responsible for up to 5 % of total environmental impacts globally [14]. In Australia, the carbon footprint of health care represents 7 % of total national emissions [15]. However, carbon footprint and unmet need have an inverse relationship, with the lowest emissions and highest unmet healthcare needs in low-income countries [16]. When we consider the disproportionate impacts of climate change in different regions, the situation is appropriately described as "possibly the largest health inequity of our time" [9]. This is because the populations most affected by climate change-related health impacts are often the least responsible for causing climate change, yet they also have the least access to healthcare needed to address these impacts.

Surgeons, and all those involved in caring for illness and injury, should be advocates for responsible environmental stewardship. As American physician Wendy Ring points out, “keeping silent on climate change is not an option for health professionals because climate change is a major health justice issue… as professionals whose mission is to promote health, this is clearly our business” [17]. Already, surgeons from multiple subspecialties have published papers arguing for reductions in the carbon footprint of their craft group [[18], [19], [20], [21]]. The aim of this perspective is to identify ways of achieving the triple goal by examining the cross-cutting intersection of literature about surgical efficacy, carbon reduction, and global healthcare inequity. It is intended to provide general recommendations, but the implementation challenges of each recommendation will vary with local context, healthcare structures, and resources. As such, this perspective is not a roadmap, but a way to provoke thought.

Systematic review of the carbon footprint of surgery and strategies to reduce it has shown that there are few high-quality studies by which to guide us [22,23]. Additionally, we felt that publications on this topic (if using a systematic approach to reference accumulation) tended to focus too much on the operating room itself [24]. We are of the opinion that the choices made by health care systems, doctors and patients regarding whether elective surgery takes place do not receive enough attention and we propose system-wide reform.

## 2 Strategies to reduce the carbon footprint of surgery whilst maintaining equitable access (Fig. 1)

### 2.1 Improve the visibility of surgical carbon footprints to our patients

Most patients give little thought to the environmental impact of the care they receive. Yet research has shown that citizens consistently rate climate change as the greatest threat faced in every country surveyed [25]. For this reason, the carbon footprint of discrete episodes of care, such as surgical operations [24,[26], [27], [28], [29], [30]], might well be of interest to patients as they make decisions about therapy.

**Fig. 1:**
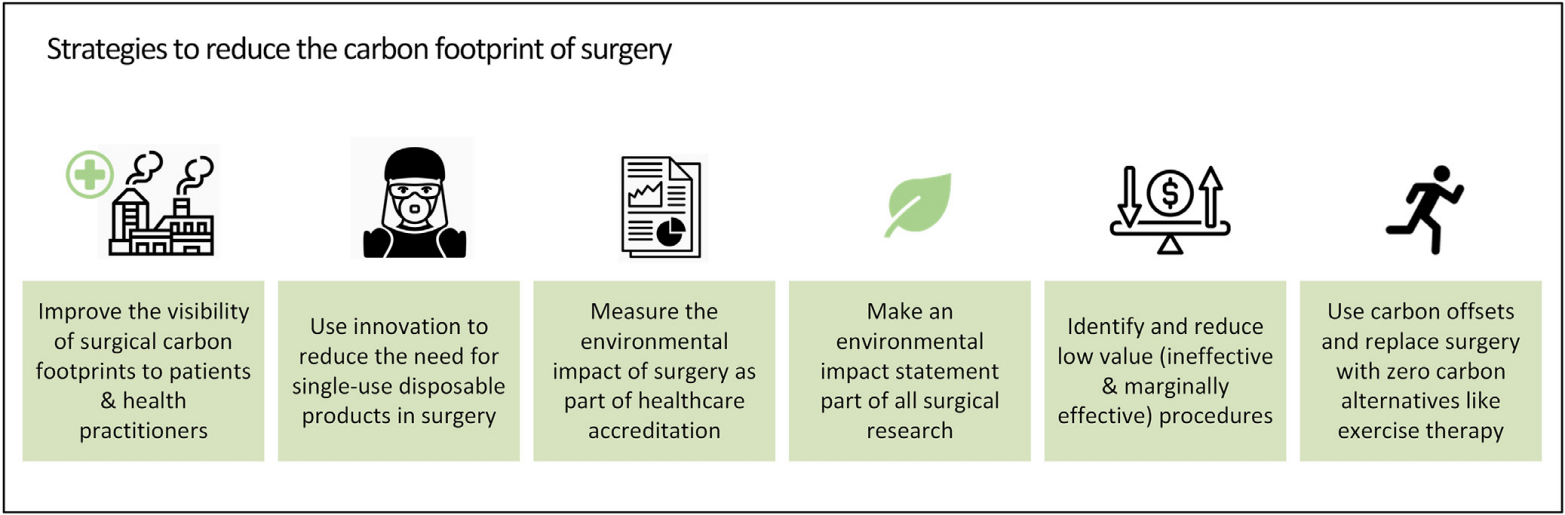
Strategies to reduce the carbon footprint of surgery.

The challenge lies in how to present understandable comparator information, and the development of intuitive metrics is an area ripe for research. For example, carbon footprints could be expressed in terms of a more familiar outputs. A typical room air conditioner will be responsible for 1368 kg CO_2_ equivalent each year [31], meaning that a single operation [32] could have the same carbon footprint of an air conditioner running through an entire summer.

An additional challenge is that the environmental footprint of many operations has not been directly measured, and it may not be feasible to do so across many thousands of different procedures carried out globally. However, it should be possible to develop generally applicable estimates of carbon equivalent per hour of theatre time in different settings, perhaps translated into an intuitive equivalent as above. This information could be offered to patients through a variety of channels, such as on a provider website, on operation-specific information sheets, or as part of an informed consent process.

It might be argued that patients would always choose individual benefits such as a robotic procedure over less proximal considerations such as climate change. However, it has been argued that the effects of climate change are themselves so immediate in the form of rising power costs, food supply disruptions, and other social determinants of health, that effective behavioural change efforts are possible through identifying co-occurring health behaviours and climate effects to patients [33]. There are programs outside of surgery such as the Clean Power Prescription programme that incentivise health behaviours and environmental justice simultaneously [34], which may be adaptable to surgical settings.

### 2.2 Drive innovation in surgical processes and supply chain

The global surgical equipment market alone has been estimated to represent almost $10 billion dollars annually and is likely to increase by as much as 10 % each year due to increasing morbidity addressable by surgery, and unmet need [35]. These markets are competitive, and there is compelling evidence that sustainability innovations are associated with increased competitive advantage. While there is little literature examining this effect in surgical equipment supply specifically, a systematic review of 100 peer-reviewed papers examining this more generally show a strong relationship between sustainability innovation and firm competitiveness. This effect seems to be mediated by increased value creation, reduced costs, and increased non-financial assets such as reputation and customer satisfaction [36,37].

One area of surgical practice in which there is likely to be considerable scope for reduction in CO_2_ emissions is the use of disposables [24]. Early literature examining the environmental impact of disposable versus reusable surgical supplies- which could be a double win for decreasing emissions in both production and disposal- has only recently begun emerging. Early analyses have tended to be unsophisticated, measuring inputs such as cost or carbon footprint, while more recent analyses incorporate life cycle analyses in keeping with rapidly advancing concepts in climate science. An example of this evolving approach are two analyses of disposable versus reusable laparoscopic instruments, one published in 2017 [38] and the other in 2024 [39]. The 2017 review notes there are too few comparative analyses to draw firm conclusions, that analyses tend to be limited to cost savings and non-standardised environmental impacts, and the advantages of reusable instruments are theoretical rather than proven [38]. In the 2024 review, four papers met the search criteria for safety, eleven for cost, and one for climate impact. The conclusion is that evidence remains extremely limited but there are clear cost and safety advantages to reusable instruments, and an “unmistakably favorable” trend towards reusable instruments for climate impact, despite the very limited data [39].

Another example of evolving climate-impact literacy is a systematic re-analysis of cradle-to-gate life cycle analyses for a range of disposable vs reusable products, including surgical drapes, surgical gowns, laparotomy pads and laryngeal mask airways [40]. The studies show a clear advantage for reusable items in terms of energy use and global warming potential ranging from just over 1:1 to 46:1 over disposables. Two studies in favour of disposables were found to contain omissions or errors, and when recalculated with the assistance of the original authors, were found to also favour reusable. It will be important for surgeons - and the organisations representing them – to develop the skills and knowledge critically appraise climate impact literature to more effectively engage with purchasing decision-makers and surgical supply manufacturers.

Innovation should be distinguished from invention. Green innovation refers to a process of applied research that aims to improve environmental performance. The processes that reduce energy use, materials, and waste tend to decrease rather than increase complexity. There is more green innovation capacity using existing, simpler techniques on wider scales compared to inventing novel equipment or techniques for narrow applications, a concept termed frugal innovation [41]. For example, a cataract procedure using phacoemulsion in the Aravind Eye Hospital in India has roughly 5 % the carbon footprint of the same operation in the UK through the cumulative savings of higher volume, reusable surgical supplies, and streamlined resterilisation processes [42]. The resource costs of techniques such as robotic surgery [43] should therefore be factored into decisions about uptake and implementation. These considerations are magnified when considering the global maldistribution of surgical resources and the ethical obligation to not increase environmental harm to those with the least likelihood of accessing novel healthcare inventions [9].

Beyond considerations of safety, cost, and environmental impact, there is a need to address implementation barriers such as user acceptability, concerns about infection control, and workflow challenges. In this area literature remains similarly limited, but a study of reusable versus disposable surgical gowns showed >79 % of users rated the reusable gowns the same or better than the disposable gown [44]. A survey of personal protective equipment (PPE) re-use during the COVID-19 pandemic, including eyewear, N95 masks, surgical masks, gloves, and gowns, showed acceptability by 85 % of end-users, with a caveat about the importance of trust and communication regarding decontamination procedures [45]. The limited number of infection control studies show comparable results between reusable and disposable instruments, but the impact on surgical workflows, such as inpatient stays and theatre delays, remain unexamined [46]. Pleasingly, with regard to health equity, a four-stage Delphi involving high- and low-income healthcare workers was able to achieve a set of climate priority interventions with 90 % acceptance to patients, although the top three priorities varied between high- and low-income respondents [47].

### 2.3 Make the measurement of the environmental impact of surgery part of healthcare accreditation processes

Institutional accreditation processes have been shown to improve health care outcomes [48], so the addition of formal environmental assessment to accreditation standards would provide positive incentives. To date, the uptake of environmental accreditation standards such as ISO-14,001 and EMAS (Eco-Management and Audit Scheme) has been slow compared to other organizational sectors. There is a paucity of reporting and public visibility about healthcare organizations that are accredited [49] despite the likelihood that this would be regarded positively by a public who rank climate change as an existential threat [25].

Continued leadership and resources will be required to address significant barriers to setting up and maintaining accreditation [2]. At present there is little literature about accreditation of surgery or surgical units specifically, and the barriers to formal ISO-14,001 or EMAS standards may prove unworkable, especially in low resource settings. Incorporation of environmental performance into existing accreditation standards may be more practical and should be prioritized. Environmental considerations in accreditation standards such as NSQHS (National Safety and Quality Health Service), JCI (Joint Commission International) and AAAHC (Accreditation Association for Ambulatory Health Care) largely relate to the internal environment of healthcare facilities [49] but could be extended to include environmentally sustainable practices in procurement, service delivery and waste management. This may not be a simple task and requires resources, time, and effort in areas in which accreditation agencies typically have little expertise, and about which even experts have not reached consensus on standards applicable to the healthcare sector.

### 2.4 Make an ‘environmental impact statement’ part of all surgical research

It has been argued that medical research should be undertaken in an environmentally sustainable way, that “we should elicit maximum knowledge and understanding for the least possible investment of resource” [50]. This approach is sensible and should be expanded: surgical research must not only assess the climate impact of research processes but also incorporate social factors like equity and the environmental impact of studied treatments [51].

Current structures of research contribute to significant waste, and this contributes to its carbon footprint. Many trials are low quality or poorly reproducible, wasting an estimated 85 % of research effort and the associated environmental impacts of carrying them out [52]. In addition, research publications are heavily skewed away from where their findings would have the greatest impact. Of 324,854 randomised controlled trials (RCTs) registered from 2010 to 2019, only 5 % were set in south Asia and 2 % in sub-Saharan African countries [53]. Similarly, there is a 1000-fold difference in DALYs (disability adjusted life years) between an emergency caesarean section, which is a lifesaving intervention often poorly accessible in low income countries, and a robot-assisted prostatectomy which carries marginal clinical benefit over a laparoscopic approach [16], and yet at the time of writing there are 110 Medline publications on the former and 3,754 publications on the latter.

To address this, there must be a renewed effort to correct global inequity in research and to fund research where it will have the greatest health impact. The ethical approval of surgical research and registration of new trials should be at least partly contingent on the potential environmental impact of the research itself, as well as the treatments studied. A justification of measures taken to avoid research waste should be required. Estimation of the carbon footprint and environmental sustainability of new procedures should become a mandatory part of outcomes reporting.

### 2.5 Identify and discourage low value surgery

The most contentious of our recommendations - yet potentially the most important – deals with the issue of procedures of such low value that their negative environmental impact might outweigh any benefit to patients. The responsibility for addressing this issue is more apparent in advanced economies, where surgery occurs at a rate higher than evidence would suggest is needed. In less resourced countries, where access to critical lifesaving surgery is still limited, surgery should not be further limited on this basis.

There are many surgical operations which have good evidence of being important for improving the health of the patient; these are considered essential, and the goal is to try to reduce the carbon footprint of each of these surgeries. On the other hand, there are some surgical operations for which there is only weak evidence for benefit [54,55]. Knee arthroscopy including partial meniscectomy is one of the most common surgical procedures and perhaps the best example of a common surgery which has failed to show superiority over both placebo surgery and comparator conservative treatment in multiple randomized control trials (RCT) [56]. As a general rule, surgery to alleviate chronic pain (which is most common in orthopaedics) is often ineffective. Other common procedures considered of dubious value on the basis of evidence include surgery for rotator cuff tears [57] and spinal fusion [58]. Some surgery is of diagnostic benefit but without evidence of much therapeutic benefit for relieving symptoms, such as laparoscopy for endometriosis [59]. Further research is required to define which surgeries in this category provide clear benefit and which do not, with the latter needing to be phased out in a transition of health systems towards net zero.

No ethical surgeon would deliberately perform surgery that has no patient benefit, so it is hard to reconcile with the thought that there may be categories of operations that are unhelpful in general. There is enough evidence to be confident that some operations provide no net benefit over placebo [54], but not yet enough evidence in many areas of surgery to be able to confidently categorise procedures into “effective” versus ineffective. Climate change – and the need for every sector including health to reduce CO_2_ emissions – does change everything: the number of surgeries currently performed multiplied by their associated emissions is not compatible with a zero-carbon economy by 2050. The lens that makes these necessary changes more palatable for surgeons is to include “carbon emissions” as a harm in the risk/benefit equation.

The definition of low-value care is itself difficult [60]. Most of the time when “low-value care” or “high-value care” terms are used, this refers to effectiveness of the intervention (over placebo or alternatives) (Table 1). Occasionally, efficiency is taken into account, meaning relative improvement per dollar spent on the intervention [60]. We strongly feel that the carbon-emissions associated with a procedure should also be routinely considered.

**Table 1:** Comparison of perspectives in considering the outcome of a randomized control trial between two different interventions.

Perspective	Key question	Focus
Traditional	Which of the two interventions gives the best results in terms of patient outcomes (based on validated outcome scores)?	Purely sees patient outcome scores as being the only item worth considering. Treats the health system as having infinite financial resources
Efficiency	Which of the two interventions gives the best results per dollar spent on the intervention?	Gives cost and patient outcomes equal weighting. Treats the planet as having an infinite carbon sink.
Holistic	Which of the two interventions gives the best results both per dollar spent and considering emissions utilized implementing each intervention?	Accepts that healthcare has both limited financial budget and limited carbon budget

For this reason, there is an urgent need to “rank” surgical procedures starting from lifesaving and life-changing operations of enormous value down to procedures whose value lies mainly in the placebo effect (Table 2). Evidence will be crucial; more randomized controlled trials (RCTs) for minimally invasive surgeries and registries for more invasive procedures are required to define this balance of value. The high-value procedures must still be performed, but in a way where carbon emissions are minimised. Low-value procedures eventually must give way, simply because business as usual is incompatible with sustaining a planet that allows human health to prosper.

**Table 2:** Healthcare provision by emissions and efficacy.

		Emission levels	
		Minimal to low	Moderate to high
Level of evidence of efficacy, taking into account cost	Strong	**Prioritise** aggressively and fund strongly e.g. exercise programs delivered by Telehealth; smoking cessation programs; vaccination	**Continue** while looking for ways to lower emissions e.g. Caesarean section for breech presentation
	Weak	**Continue** while funding further studies regarding efficacy e.g. physiotherapy for arthritis	**Review** until lower emission alternative is available, or evidence demonstrates sufficient benefit against the costs and harms of associated emissions
	No evidence of efficacy OR evidence of not being effective	**Review** until evidence available, especially regarding avoidable harm	**Restrict or defund** e.g. knee arthroscopy for degenerative knee symptoms [47]

Making the hard decisions about low-value surgery will be a test of surgical leadership and interprofessional collaboration. Surgeons are already trained to incorporate equity at a local level into treatment decisions, such as through scoring systems to avoid treatment bias or in targeted health messaging to higher risk groups. Expanding this to consider equity at a global and climate level will require a significant cultural shift. Continuing to perform low-value carbon-intensive procedures that are unsupported by evidence will be increasingly incompatible with the critical goal of reaching a carbon neutral society by 2050, and out of step with parallel efforts occurring in anaesthesia and critical care [61].

Surgery has always been about action, not inertia. We have the capacity to make the necessary changes and must grasp this leadership challenge.

### 2.6 Use carbon offsets where possible

The sheer volume of surgery undertaken globally, and the environmental impact of operations and perioperative care, means that changing to ‘green’ surgery will be a monumental undertaking that must balance the needs of patients with the health of the planet. There are no easy pathways to low-carbon environmentally sustainable surgery.

While we deal with this transition – perhaps the greatest transition in surgical care since the development of asepsis – one potential strategy is the use of ‘carbon offsets.’ This is a way of paying for a reduction in carbon emissions elsewhere to compensate for emitted greenhouse gases. Carbon offsets are controversial because higher-income countries have more ability to purchase offsets, while demand to provide offsets can disrupt subsistence activities in low- and middle-income countries, such as through planting trees in areas which might otherwise be used for agriculture [62]. In addition, organisations often purchase carbon offsets at the lowest ‘cost’ which is often artificially low compared to the actual cost. An example is the very low price offered by airlines to ‘offset’ travel compared to the estimated median social cost of carbon (the cost to humanity of one tonne of CO_2_) of USD$417/tCO_2_ [63]. Therefore, while the use of carbon offsets is one way of taking urgent interim action to reduce the carbon footprint of healthcare in developed countries, this is only a temporary measure until such time that sustainable surgery can be achieved [64].

## 3 Conclusion

Surgery is a major source of CO_2_ emissions [65]. Operating rooms are resource intensive and up to six times more energy intensive than other areas of a modern hospital [66]. A single operation can be associated with emissions of 814 kg CO_2_ equivalent [32], more than the total *annual* emissions of a person in Papua New Guinea or Zimbabwe [67] and such estimates do not consider the additional environmental effects of adjuvant procedures such as pathology testing [68]. A review of the carbon footprint of minimally invasive surgery (MIS) in the United States concluded that “if MIS in the United States was considered a country, it would rank 189th on the United Nations’ 2008 list of countries’ carbon emissions per year” [69]. Robotic surgery generates up to 38 % more carbon emissions than non-robotic laparoscopic surgery, and 77 % more than open surgery [43]. Even surgery-related activities such as perioperative clinics, surgical teaching, and conferences have been analyzed and found to have a substantial carbon footprint [27,70].

As a professional group, surgeons have an ethical obligation to provide the highest quality care to patients, yet they also have a broader obligation to enhance the health of communities in which they live and practice. Writing on the importance of surgical leadership in the *British Journal of Surgery*, Rothmund [71] reminds us that “although the virtues of knowledge, technical excellence, research ability and teaching skill are far from obsolete, the ideal surgical leader of today requires much more… The role of a healthcare advocate is an important one for surgical leaders.” For surgery to maintain its leadership role in healthcare and directly impact patient care quality [72], it must address the climate impact associated with surgical practices.

Surgeons do not act alone, of course. They are embedded in systems, and there will need to be corresponding actions at every level throughout the complex healthcare landscape. The specific stakeholders will depend on local contexts globally but may include, for example, hospital administrators, insurers, regulators, legislators, and professional associations. The advocacy suggested by Rothmund can take many forms, such as through encouraging professional societies to develop climate change policies and embed sustainable practices in their work, engagement in education and media, and amplifying the voices of under-represented populations. Surgeons can lobby or support renewable energy at hospital, state, and national level. They can provide remote options more routinely for conferences, and work towards less fragmented work patterns to decrease commuting between sites. The scale and complexity of the problem demands a multifactorial approach, and the need for rapid action means data to inform action will often be partial and post-hoc. Surgeons, who train to perform in complex evolving clinical situations, are well placed to apply these skills to environmental change.

High quality surgery is critical to modern health care and access to surgery is one of the most important ways of improving global wellbeing [73]. However, modern healthcare in general, and surgery in particular, is a substantial contributor to global greenhouse gas emissions. Surgeons have a reputation for balancing the best available evidence with the needs of individual patients to provide compassionate care – to make the lives of their patients better. Surgical leadership will be required in great measure to make sure that the surgery of the future is sustainable, equitable, and does the greatest good with the least harm. The stakes are high, and time is running out: it is time that the harms of surgery are broadened to include the harms of inequity and harms to the planet.

## Funding statement

This research did not receive any specific grant from funding agencies in the public, commercial, or not-for-profit sectors.

## CRediT authorship contribution statement

**Rhea Liang:** Writing – review & editing, Writing – original draft, Visualization, Investigation, Formal analysis, Data curation, Conceptualization. **John W. Orchard:** Writing – review & editing, Writing – original draft, Visualization, Formal analysis, Data curation, Conceptualization. **Stephen J. Robson:** Writing – review & editing, Writing – original draft, Methodology, Formal analysis, Data curation, Conceptualization.

## Declaration of competing interest

The authors declare that they have no known competing financial interests or personal relationships that could have appeared to influence the work reported in this paper.
